# Peer mentoring experience on becoming a good doctor: student perspectives

**DOI:** 10.1186/s12909-020-02408-7

**Published:** 2020-12-07

**Authors:** Mohd Syameer Firdaus Mohd Shafiaai, Amudha Kadirvelu, Narendra Pamidi

**Affiliations:** Jeffrey Cheah School of Medicine and Health Sciences, Monash University Malaysia, Jalan Lagoon Selatan, Bandar Sunway, 47500 Subang Jaya, Selangor Malaysia

**Keywords:** Peer mentoring, Peer teaching, Peer to peer teaching, Medical student, Undergraduate, Cohort study

## Abstract

**Background:**

PASS is a peer-led structured academic mentoring program designed to provide academic assistance for new students in their transition from college to university studies and also for students struggling in certain units. This study aims to establish acquired skills by peer leaders associated with peer-led mentoring via the PASS program, and to explore the role played by these acquired skills in their journey to become a successful doctor.

**Methods:**

Study participants were forty selected second-year undergraduate medical students at Monash University Malaysia with commendable examination results. Validated pre-test and post-test questionnaires were administered to explore changes in the level of communication, leadership, professional, and pedagogical skills before and after participation in peer mentoring program. Qualitative analysis of focused group interviews was performed by an independent investigator to identify how the skills developed as a peer mentor may help with becoming a good doctor. Major themes were identified with the thematic-analysis approach.

**Results:**

Thirty-eight students completed the pre-test and post-test questionnaires. Peer leaders reported improvement in oral and written skills for teaching; increased confidence to give constructive feedback; better stress management; efficient time management; improved interpersonal skills; and enhanced problem-solving and critical thinking capabilities. Eight major themes were identified from the interview and peer leaders reported positive experience of working in diverse environments and shouldering of responsibilities.

**Conclusions:**

Peer-led mentoring provides a good opportunity for medical students to shoulder responsibilities as a leader and offers an experience of managing a team of their peers and juniors which in turn may enhance their communication, interpersonal, and leadership skills.

**Supplementary Information:**

The online version contains supplementary material available at 10.1186/s12909-020-02408-7.

## Background

The year was 55 BC when the esteemed Roman statesman, Cicero first coined the Latin term, ‘Docere’, which beautifully described the role to teach, to delight and to move. It wasn’t until the thirteen centuries when the world’s oldest university, i.e. the University of Bologna in medieval Europe had first conferred a doctorate degree, which bestowed the holder the rights to teach with the title doctor [1, 2]. Today’s in this modern era, each and every medical doctor plays a role in teaching, be it their peers, their mentees, or their patients.

Peer Assisted Study Sessions (PASS) is a peer-led structured academic mentoring program designed to provide academic assistance for new students in their transition from college to university studies and also for students struggling in certain units. PASS is an Australian adaptation of Supplemental Instruction which was developed by Deanna Martin at University of Missouri with aim to increase students’ success in difficult courses [3]. In our medical school, it is delivered by accomplished senior year medical students to junior medical students on a weekly basis after rigorous selection and training. Friendly peer leaders are empowered with guided support to impart their knowledge and experience, and motivate mentees to do better with coursework.

Internationally, peer-led mentoring program has become a widespread phenomenon across all fields of expertise and scale of organization including the healthcare field [4, 5]. Amongst the Fortunes 500 companies, more than 70% of them are implementing peer mentoring programs [6]. Smaller institutions such as startups, on the other hand are working to provide environments where mentoring will thrive organically [7]. The structured mentoring program is currently regarded as the gold standard in mentoring because the mentor is first trained to mentor which then results in improvement of their mentoring knowledge, skills, and demeanors at mentoring [8, 9].

At present, literatures on intricacies of relational dynamic between mentor and mentee, the performance of mentor evaluation, and satisfaction of mentee are in abundance [10, 11]. The raison d’etre for this burgeoning phenomenon is perhaps due to the positive outcomes from multiple studies associated with peer mentoring. Studies have reported positive experience amongst former peer leaders, positive feedback from mentees, opportunities for development of leadership skills, increased sense of engagement in the community, and the unique traits of the new generation of workforce which demand changes in doctrine for their training [12–14].

Current literature on experience of peer leaders are focused on measuring three key components of mentoring which are role-modelling, psychosocial support, and vocational support [15]. Results from these studies have been overwhelmingly positive. Another common theme being researched on is the intricacies of relational dynamics between mentor and mentees in a peer-led mentoring program. Many a time, mentees reported difficulties regarding several aspects of relational challenges which often are communication and interpersonal in nature. Some examples include difficulties in confronting hurdles and managing expectations of mentor [10].

In the current era, doctors are expected to be an effective communicator with good interpersonal skills to navigate through the complexity of the healthcare system and patient-doctor relationship. Living up to the Latin root word, docere, a doctor in this ever-changing world is also expected to be a good teacher and mentor [16, 17]. However, there is inadequate data on the association of skills gained as a peer mentor and its relevance to their future practice as a medical professional.

This study aims to establish the skill set acquired by peer leaders during peer mentoring and to explore how these skills will be helpful in their journey to become a successful medical practitioner.

## Methods

### Setting

The study was done at Jeffrey Cheah School of Medicine and Health Sciences, Monash University Malaysia over a period of 2 years from 2018 to 2019.

### Characteristics of study participants

Study participants were undergraduate second-year pre-clinical medical students who were selected through an interview for their excellent communication and interpersonal skills, and have achieved excellent results in all course-related examinations with a ranking of upper third quartile in their cohort of students. This study was approved by the Monash University Humans Research Ethics 2010 Committee, Approval No. 17004.’

### Description of materials

The pre-test questionnaire is a validated (Cronbach’s a 0.876) self-report measured on a 5-point Likert scale and included 12 items with focus on establishing the baseline level of communication skill and pedagogical skill (see Additional file 1); the post-test questionnaire is a validated (Cronbach’s a 0.797) self-report measured on a 5-point Likert scale and included 12 items with focus on establishing the post-intervention level of communication skill and pedagogical skill (see Additional file 2).

Both questionnaires utilized a 5-point Likert scale to measure agreement of the participant for each item, i.e. 5 is strongly agree, 4 is agree, 3 is neutral, 2 is disagree and 1 is strongly disagree. In addition to the questions related to their communication skills and pedagogical skill, both questionnaires also collected demographic information (age and gender).

The structured interview consisted of 8 open-ended questions with a focus on exploring whether or not participation in the PASS program as peer leaders helped in developing skills required to become a successful doctor in the future from the participant’s point of view (Additional file 3).

### Study processes, interventions, and comparisons

Our study has utilized a mixed-study approach, i.e. quantitative survey and focus group discussion to achieve the objectives. This approach allowed for triangulation of data between established individual domains of skills obtained from literatures through participation in peer-mentoring program, and the real context for individual domains of skills identified from the peer leaders who have participated in our PASS program.

To objectively identify individual domains of skills based on existing literatures which the peer leaders would have obtained through their participation in the PASS program, we have utilized a combination of pre-test and post-test questionnaires. Baseline skills possessed by the peer leaders prior to participation in the peer mentoring program were established via the pre-test questionnaire. Post-test skills possessed by the peer leaders after participation in the peer-mentoring program were identified via the post-test questionnaires.

Prior to the training, participants were asked to complete a pre-test questionnaire. Following which, the two-day PASS leaders training is conducted by a certified trainer-supervisor for PASS program. The training is focused on essential elements of PASS program, i.e. planning for a PASS session, facilitating a PASS session, and handling administrative work as a PASS or peer leader. On completion of the training, peer leaders in pairs are then assigned to their respective groups of juniors for one semester. Mentees in each group were facilitated by their peer leaders in an 1 h weekly based integrated learning session through problem-solving and active discussion about various topics in anatomy, biochemistry, physiology and clinical skills etc. At the end of the session, mentees were expected to have better understanding about the subject matter after being tutored by their peer mentors. After 12 weeks, the leaders were asked to complete a post-test questionnaire.

One year after the completion of the 12 weeks program, we then conducted a focus group discussion with the peer leaders while they are in their third year of study which is also their first year in clinical setting. Reason for this is to further enhance the confidence in our findings through focus group discussion with the aims: to establish missing domains of skills obtained through peer mentoring which we may have not included in our validated questionnaires, to correlate our findings with those established domain of skills in the current literatures via thematic coding and classification of open-ended responses from our focus group discussion, and to as completely as possible establish a set of skills obtained by peer leaders through peer mentoring [18].

In order to triangulate the data, we have used multiple types of triangulation to enhance reliability of our findings: 1) method triangulation: data collected from focus group interview were triangulated against our questionnaires; (2) data triangulation: thematic coding and classifications of open-ended responses were independently done by two coders and reviewed by the investigators to come about with the themes and sub-themes of skills gained from participating in PASS program [19, 20].

### Statistical analysis

Demographic data and responses to quantitative questions are presented as means and proportions. The normality test is performed using Shapiro-Wilk Test. A paired T-test is used to compare between items in post-test and pre-test group. Statistical analysis is performed by using IBM SPSS Version 22. The general level of significance is fixed at 0.05.

Qualitative data was analyzed by using thematic-analysis approach to search for salient points of common experiences. First, a recorded interview was done which was transcribed and fed into an analytical software, i.e. NViVo 12.6.0. Relevant quotes were highlighted and grouped into a suitable thematic category. Next, multiple thematic categories which described a specific version of similar experience were combined to create a more inclusive thematic category. Additionally, subcategories that stood on their own but described another theme were then created. Finally, thematic categories were checked to ensure all quotes contained within them fit well into their assigned categories.

## Results

### Informant sampling and respondent demographics

Of the 40 peer leaders who were sent questionnaires, 38 responded. Informant characteristics are summarized in Table 1. Sixteen of the participants were male (40.0%), twenty-three were female (57.5%) and one participant did not identify the gender (2.5%). The mean age was 20.9 years old. 15 peer leaders who consented to participate in focus group discussion were interviewed a year later.

**Table 1:** Demographics of Participants

	Gender	Frequency	Percentage
Gender	Male	16	40.0
	Female	23	57.5
	Not identified	1	2.50
Year of Study	2	19	47.5
	3	21	52.5

### Results of quantitative analysis

#### Communication skills

On completion of PASS program, peer leaders reported a mean score increase of 0.667 (95% CI 0.155–0.12; *p* > 0.05) in oral and written skills to engage with the junior students and peers with mean score of 4.08 ± 1.13 at baseline; 0.778 (95% CI 0.349–1.207; p > 0.05) mean increase in their skills to develop interaction and collaborations amongst students with a mean score of 4.00 ± 1.12 at baseline; 0.794 (95% CI 0.512–1.076; *p* < 0.05) mean score increase in their abilities to provide constructive feedback on student learning with baseline score of 4.21 ± 0.808.

#### Leadership skills

Peer leaders reported a mean score increase of 0.824 (95% CI 0.508–1.139; *p* < 0.05) in their working in team and leadership skills after completion of the PASS program with a baseline mean score of 4.29 ± 0.68.

#### Personal attributes

Peer leaders reported a mean score increase of 0.706 (95% CI 0.263–1.148; *p* < 0.05) in their stress and time management skills with baseline mean score of 4.12 ± 0.145 after completion of PASS program; 0.706 (95% CI 0.455–0.957; *p* < 0.05) mean score increase in their abilities to plan and organize a teaching session as per the scheduled timeline for peer teaching session with baseline mean score of 4.44 ± 0.561.

#### Pedagogical skills

Peer leaders reported a mean score increase of 0.765 (95% CI 0.454–1.075; *p* < 0.05) in their interpersonal and critical thinking skills with baseline mean score of 4.21 ± 0.641 after completion of PASS program; 0.778 (95% CI 0.349–1.207; *p* < 0.05) mean increase in their ability to create an effective learning environment with baseline score of 4.00 ± 0.906; 0.471 (95% CI 0.196–0.745; *p* > 0.05) mean score increase in their abilities to learn new skills with baseline score of 4.38 ± 0.551; 0.912 (95% CI 0.610–1.124; *p* < 0.05) mean score increase in their abilities for problem-solving and innovative thinking in peer teaching session with baseline mean score of 4.21 ± 0.101; 1.235 (95% CI 0.913–1.557; *p* < 0.05) mean score increase in their skills to facilitate teaching sessions with baseline mean score of 4.50 ± 0.707; 0.912 (95% CI 0.586–1.237; *p* < 0.05) mean score increase in their abilities to develop independent learning amongst their students with baseline score of 4.06 ± 0 (Table 2).

**Table 2:** Student’s T-test comparing mean differences of post-test score and pre-test score

		Paired Differences					t	df	Sig. (2-tailed)
		Mean	Std. Deviation	Std. Error Mean	95% Confidence Interval				
					Lower	Upper			
Pair 1	1. My oral and written skills to engage with students and peers have improved - 1. I have the effective oral and written skills to engage with the students and peers	.667	1.512	.252	.155	1.178	2.646	35	.012
Pair 2	2. My ability to create an effective learning environment have improved - 2. I have the abilities to create an effective learning environment	.778	1.267	.211	.349	1.207	3.682	35	.001
Pair 3	3. My skills to develop interaction and collaborations amongst the students have improved - 3. I have the skills to develop interaction and collaborations amongst the students.	.824	.904	.155	.508	1.139	5.315	33	.000
Pair 4	4. My teamwork and leadership skills have improved - 4. I have the effective teamwork and leadership skills to manage the student groups and peer	.735	.710	.122	.488	.983	6.042	33	.000
Pair 5	5. My interpersonal and critical thinking skills have become better - 5. I have the interpersonal and critical thinking skills	.765	.890	.153	.454	1.075	5.012	33	.000
Pair 6	6. My stress and time management skills have improved - 6. I have the effective stress and time management skills	.706	1.268	.217	.263	1.148	3.246	33	.003
Pair 7	7. My ability to welcome and learn new skills has enhanced - 7. I have the ability to welcome and learn new skills	.471	.768	.135	.196	.745	3.484	33	.001
Pair 8	8. My innovative thinking and problem-solving abilities in peer teaching have strengthened – 8. I have the innovative thinking and problem-solving abilities to make an effective peer teaching/facilitation session	.912	.866	.148	.610	1.214	6.141	33	.000
Pair 9	9. My skill on planning and organising the teaching sessions as per the timeline is better - 9. I can plan and organize the teaching sessions as per the timeline	.706	.719	.123	.455	.957	5.725	33	.000
Pair 10	10. My learning strategies/skills to facilitate the teaching sessions have improved - 10. I have the effective learning strategies/methods to facilitate the teaching sessions effectively	1.235	.923	.158	.913	1.557	7.803	33	.000
Pair 11	11. My teaching strategies to develop independent learning amongst the students have improved - 11. My teaching strategies will help to develop independent and collaborative learning amongst the students	.912	.933	.160	.586	1.237	5.697	33	.000
Pair 12	12. My ability to provide constructive feedback on student learning have improved - 12. I have the ability to provide constructive feedback on student learning	.794	.808	.139	.512	1.076	5.729	33	.000

### Results of qualitative analysis

The comments and stories related by student leaders in the structured interview were categorized into themes pertaining to how PASS program helps with preparing to become a successful medical practitioner. Two major themes were identified which are personal growth and professional growth.

### Personal growth

Four sub-themes consisting of individual skills development were identified. These skills are namely communication skills, leadership skills, learning skills, and pedagogical skills. They are consistent with the results from our quantitative analysis where peer leaders reported improvement across communication skills, leadership skills, and pedagogical skills.

#### Communication skills

The majority of peer leaders identified the ability to connect and reciprocate with peers and students as an essential skill to conduct a successful PASS session which is pivotal to become a successful doctor. In keeping with the results from quantitative analysis, peer leaders have elaborated further on communication skills gained through the PASS program. These skills consisted of rapport building with students and peers, giving and receiving constructive feedback, responding to non-verbal cues, and setting expectations .

A female medical undergraduate on the topic of building rapport stated:

*“It definitely helps us because we now know how to build rapport with our peers; in clinical practice we build rapport with our patients. This will enable us to get the information across quickly.”*

Another male medical undergraduate remarked on giving feedback:

*“I tend to be nice to a person when giving feedback. I learnt via PASS that giving good feedback, a frank one is important. That is more beneficial. When it comes to clinical practice, I would need to be truthful and critical with my patient.”*

A female medical undergraduate commented on setting expectation:

*“In order to create the most effective learning environment, I would need to know what their needs and expectations are....I had to put myself in their shoes. This is important when it comes to understanding a patient, hence the importance for patient-doctor relationships.”*

#### Leadership

Peer leaders identified good leadership as the key to stewarding the teaching session successfully. Other findings which have surfaced from the focus group interview are maturity and confidence in interacting with other students which are important elements of good leadership.

A male medical undergraduate who identified as an introvert remarked on instilling confidence as a key to good leadership:

*“I feel that it is important to be confident and also to portray confidence when it comes to patient practice. This is true when handling the juniors, we need to portray confidence with the juniors so that we can instill confidence for them to have active discussion with them.”*

A female medical undergraduate remarked on maturity as part of the process of being a leader:

*“When I started, I was shy and ashamed at times when I didn’t know the answers for certain things … But after some time, I realized that learning is not a one-way learning process. While teaching, I was learning too of what I didn’t know... So, I will find the answer and share with the rest of them.”*

#### Learning

Another key skill which the peer leaders have identified through focus group interviews but not present in the quantitative analysis part is the learning skills gained via PASS program. Peer leaders described three keys to learning which are resourcefulness, active learning and collaborative learning.

A male medical undergraduate who was in charge of the peer leaders for PASS program commented on resourcefulness:

*“It does tickle our brain on how to answer those questions posed by our juniors from a different perspective. It requires us to stay updated with the latest resources. In future, we will do the same in the management of our patients too.”*

A female medical undergraduate who assisted the head of peers for PASS program remarked on collaborative learning:

*“PASS does help us a lot because during PASS, we can discuss things we don’t understand with our juniors.”*

#### Pedagogical

Peer leaders identified two important attributes from the experience of teaching junior students through which are prerequisite to successful active learning and teaching sessions. These attributes are creativity and critical thinking which were honed further via participation in the PASS program.

A tech-savvy male medical undergraduate remarked:

*“We learned that we have to make our session more interactive, e.g. via active discussion and participation such as Kahoot.”*

A female medical undergraduate reaffirms her challenging experience to hone critical thinking skills for PASS remarked:

*“It does really help me in improving my critical skills because we conduct the session from different perspectives. It reflects back on my clinical practice where it helps me to think holistically as a patient -treat the patient vs treat the disease.”*

### Professional growth

Four sub-themes were identified. These themes are consistent with the personal attributes such as time management skill and administrative skill gained by peer leaders through participation in the PASS program as reported in the quantitative analysis part. Further elaborations were made by the peer leaders in the focus group interview regarding these themes.

#### Administrative

Peer leaders identified skills gained from administrative duties of being a peer leader as one of the keys to success as a doctor as well and would increase the threshold to burn out in future. These skills include managing schedules by coordinating timing between students and tutors to organize a teaching session, and juggling between responsibilities both as a student-teacher and a peer.

A male medical undergraduate peer leader noted on administrative duties as a peer leaders:

*“Management skill is something we have gained, and I feel that it will allow us to manage ourselves better hence not to burn out.”*

#### Experience

Experience is an important aspect of PASS program. This is another theme that surfaced from the focus group interview but is not present in the quantitative analysis part. Peer leaders reported experience in conflict resolution, opportunities for learning and observing different presentation styles, complexity of teamwork and challenges of working with diversity amongst the top experience of being a peer leader. .

A female medical undergraduate who is also a highly-decorated scholar reported:

*“By teaching our juniors, I feel that it does help me with my presentation skills. . This is because presentation is a big part of being a medical student, and a doctor. During a continuing medical education session or a medical conference, we would need to do a presentation and read up on the topic as well. This is what I have experienced while being a peer leader.”*

Another gender non-binary student identified teamwork as an important experience of being a peer leader:

*“I think it has made us realize that to share responsibilities between the peer leaders is important.”*

An international male medical undergraduate noted on working with diversity:

*“I have to communicate with my juniors as well who are of different backgrounds and culture, thus PASS program helps me in this aspect.”*

#### Responsibility

Through the focus group interview, a new theme is identified which is not present in the quantitative analysis part. Peer leaders have identified four aspects of being a responsible peer leader which will shape a good character of a responsible doctor which are to do adequate preparation, honoring commitment, honesty as the best policy and lifelong learning.

An female medical undergraduate reported:

*“Not only that we have to prepare for discussion, but we have to also be responsible and answerable to what we are going to tell our juniors because we cannot just blurt out everything. “*

Another male medical undergraduate noted:

*“We wanted to do quality work and be committed to our juniors’ learning. It made us more responsible.”*

#### Time management

Peer leaders reported three essential skills related to time management which have been acquired and honed through participation in the PASS program. This is in keeping with the results from our quantitative analysis. These skills are prioritizing tasks, punctuality, and having a work-life balance.

A female medical undergraduate commented on prioritizing of tasks:

*“We learned how to manage our time effectively and to weigh the priority of the topics to focus on for our juniors and our own revision.”*

Another male medical undergraduate spoke of punctuality:

*“Our juniors taught us to be on time. I feel that it is an important trait as a successful doctor.”*

And a female medical undergraduate further added:

*“With PASS, we have to manage our time well to ensure we get up to date with our tasks and that everyone is on track as well. This would make an important and good trait of a doctor.”*

## Discussion

In our study, peer mentoring experience contributed to the development of skills and attributes that are considered essential to become a good doctor. The acquired skills from participation in peer mentoring program are consistent with the core clinical competencies of a good doctor, i.e. competent interpersonal and communication skills which result in effective bilateral exchange of information between doctor and patient; practicing professional attributes and demeanors such as empathy, compassion, calmness, attentiveness, adaptability, passion, confidence, and humility [17].

Findings from our study are consistent with findings from previous studies concerning peer leaders in medical education. These studies found that with participation in peer-led mentoring program, peer leaders are more organized and better at time management; they are ready to negotiate challenges and utilize resources to support their work; they feel driven from healthy peer pressures of being with crème de la crème pool of peer leaders; their learning and pedagogical skills are enhanced; they feel more inclusive in the community and contempt with their contribution at mentoring their juniors [21–23].

We did not find significant differences after peer mentoring experience in written and oral communication skills as well as interaction and collaboration skills amongst our peer leaders. It could be because our cohort of peer leaders were chosen for the program based on their strong communication and interpersonal skills.

Arguably, the population of medical students and junior doctors in years to come would largely be made up of millennials. Such a generational shift would require a colossal shift in the doctrine for their training as well [24, 25]. Millennials are known for being confident, narcissistic, collective minded, and diverse in thinking [26]. They value personalized learning experience, mentoring by seniors, working in team and incorporation of technology in their work [27]. In order to cater to their unique learning needs and personality traits, the learning approach should work around their values.

In PASS, our peer leaders are empowered with a clear set of responsibilities and skills needed to conduct a teaching session through our structured peer mentoring program. They are given the freedom to plan and conduct sessions amongst themselves which provide a room for them to breathe amidst their own tight schedule at medical school. Topics of choice for each session are mutually decided and agreed to be beneficial for their own learnings and the mentees as well. This form of active learning with clearly defined structure is proven effective [28]. The opportunities to teach, engage and contribute back to their community are also aligned with the values and attributes of millennials. Many studies have cited these values to be the deciding factor for many millennial peer leaders to join such a program in spite of their tight schedule [23].

There are limitations to our study. First, our study relies exclusively on self-reported measures by peer leaders of their own experience. There is no measure of peer leader’s performance from the perspective of their mentees. Second, our peer leaders are only representative of a single centre and their experiences are not reflective of the experience of peer leaders from other centres where there are variations on the doctrine of the peer-led mentoring session as well as its benefits and drawbacks. Third, we have made the assumptions that for data with sample size less than 200, and where the group variances are equal if the ratio of the largest and smallest variances are less than 3, there is no significant difference between parametric and non-parametric analysis based on literature. These assumptions are made to justify the usage of T-test for the analysis of the Likert Scale data that we have obtained [29, 30]. Forth, there is no intergenerational interaction in our peer mentoring program. Our PASS leaders and their mentees are both millennials, and thus there is no intergenerational interaction between mentor and mentee which could result in different experiences. Lastly, our population of peer leaders consist exclusively of undergraduate medical students -had postgraduates students included as well, the opinions may be different.

Future research in this field of peer-led mentoring should include diverse populations of undergraduate and post-graduate medical students from multiple centres to get the bigger picture of their experience as peer leaders. Feedbacks from mentees regarding their peer leader’s performance throughout the PASS program should also be analyzed and compared against self-report measure by peer leaders themselves as this will allow for self-report bias to be eliminated from the final study. Postgraduate medical students should also be included in the pool of peer leaders to increase the diversity of the study population.

## Conclusion

Our study has shed light on acquired skills and attributes associated with peer-led mentoring and how peer leaders perceived these qualities to be useful in their journey as a future doctor. We conclude that peer-led mentoring provides good opportunity for medical students to shoulder responsibilities as a leader and experience managing their peers and juniors which in turn enhance their communication, interpersonal, pedagogical and leadership skills. These qualities are in concordance with the requirement of future medical workforce which amongst others require healthcare workers with the right skills to provide the right care, in the right place, at the right time, and with competent leadership skills, communication skills and the ability to work within a team [31].

## Supplementary Information

**Additional file 1.** re-Test Questionnaire. A set of pre-test questionnaire with twelve validated questions on a five-point Likert scale.

**Additional file 2.** Post-Test Questionnaire. A set of post-test questionnaire with twelve validated questions on a five-point Likert scale.

**Additional file 3.** List of Questions for Structured Interview. A set of eight questions for structured interview.

## Data Availability

The datasets generated and/or analysed during the current study are available in the Open Science Framework repository, DOI 10.17605/OSF.IO/CSNQH and available via https://osf.io/csnqh/

## Abbreviations

IBM SPSS: International Business Machines Statistical Product and Service Solutions
PASS: Peer Assisted Study Session
