# Supplementary material for: Peer mentoring experience on becoming a good doctor: student perspectives
Source: BMC Med Educ. 2020 Dec 7;20:494. doi: 10.1186/s12909-020-02408-7 (PMC7720515; doi:10.1186/s12909-020-02408-7)
Supplement: Supplementary file 1 — Additional file 1. re-Test Questionnaire. A set of pre-test questionnaire with twelve validated questions on a five-point Likert scale. [file 12909_2020_2408_MOESM1_ESM.pdf]

Pre-Test Questionnaire

Question	Likert Scale				
	5<br>Strongly Agree	4<br>Agree	3<br>Neutral	2<br>Disagree	1<br>Strongly Disagree
1. I have the effective oral and written skills to engage with the students and peers					
2. I have the abilities to create an effective learning environment					
3. I have the skills to develop interaction and collaborations amongst the students					
4. I have the effective teamwork and leadership skills to manage the student groups and peers					
5. I have the interpersonal and critical thinking skills					
6. I have the effective stress and time management skills					
7. I have the ability to welcome and learn new skills					
8. I have the innovative thinking and problem-solving abilities to make an effective peer teaching/facilitation session					
9. I can plan and organize the teaching sessions as per the timeline					
10. I have the effective learning strategies/methods to facilitate the teaching sessions effectively					
11. My teaching strategies will help to develop independent and collaborative learning amongst the students					
12. I have the ability to provide constructive feedback on student learning					
