# Supplementary material for: Peer mentoring experience on becoming a good doctor: student perspectives
Source: BMC Med Educ. 2020 Dec 7;20:494. doi: 10.1186/s12909-020-02408-7 (PMC7720515; doi:10.1186/s12909-020-02408-7)
Supplement: Supplementary file 2 — Additional file 2. Post-Test Questionnaire. A set of post-test questionnaire with twelve validated questions on a five-point Likert scale. [file 12909_2020_2408_MOESM2_ESM.pdf]

Post-Test Questionnaire

Question	Likert Scale				
	5<br>Strongly Agree	4<br>Agree	3<br>Neutral	2<br>Disagree	1<br>Strongly Disagree
1. My oral and written skills to engage with students and peers have improved					
2. My ability to create an effective learning environment have improved					
3. My skills to develop interaction and collaborations amongst the students have improved					
4. My teamwork and leadership skills have improved					
5. My interpersonal and critical thinking skills have become better					
6. My stress and time management skills have improved					
7. My ability to welcome and learn new skills has enhanced					
8. My innovative thinking and problem-solving abilities in peer teaching have strengthened					
9. My skill on planning and organising the teaching sessions as per the timeline is better					
10. My learning strategies/skills to facilitate the teaching sessions have improved					
11. My teaching strategies to develop independent learning amongst the students have improved					
12. My ability to provide constructive feedback on student learning have improved					
