# Supplementary material for: Peer mentoring experience on becoming a good doctor: student perspectives
Source: BMC Med Educ. 2020 Dec 7;20:494. doi: 10.1186/s12909-020-02408-7 (PMC7720515; doi:10.1186/s12909-020-02408-7)
Supplement: Supplementary file 3 — Additional file 3. List of Questions for Structured Interview. A set of eight questions for structured interview. [file 12909_2020_2408_MOESM3_ESM.pdf]

List of Questions for Structured Interview

**Question**
1. Does the PASS leaders experience will help in working with diverse group of people?
2. The experiences in PASS sessions prepared me for work readiness and commitment to lifelong learning for patient practice.
3. Does the management skill acquired will help in community management or engagement?
4. The interpersonal and critical thinking skills acquired will help in clinical practice?
5. Does the skill acquired to create an effective learning environment will help in patient interaction/management?
6. Does the skills acquired to provide an effective and constructive feedback will help in patient practice?
7. Does the improved communication skills will help in handling/managing the patient and peers?
8. Any additional skills required?
